# Epstein–Barr Infection, Hodgkin’s Lymphoma, and the Immune System: Insights into the Molecular Mechanisms Facilitating Immune Evasion

**DOI:** 10.3390/cancers17091481

**Published:** 2025-04-28

**Authors:** Eleni Tsotridou, Emmanouel Hatzipantelis

**Affiliations:** Children’s and Adolescents’ Hematology Oncology Unit, 2nd Department of Paediatrics, AHEPA University General Hospital, School of Medicine, Faculty of Health Sciences, Aristotle University of Thessaloniki, St. Kyriakidi 1, 54636 Thessaloniki, Greece; hatzip@auth.gr

**Keywords:** Epstein–Barr virus, Hodgkin’s lymphoma, immune evasion, innate immunity, adaptive immunity, immune checkpoints, tumor microenvironment, immunotherapy

## Abstract

Epstein–Barr virus (EBV) constitutes a very common pathogen and a well-characterized carcinogen. EBV has the ability to establish a chronic latent infection, during which only a subset of the viral genes is expressed. EBV is implicated in multiple malignancies, including Hodgkin’s lymphoma (HL). HL mainly affects adolescents and young adults and has an overall favorable prognosis. However, relapsed or refractory disease still poses a therapeutic challenge. EBV does not only induce malignant transformation but also hinders the detection and clearance of the neoplastic cells by the immune system. The proteins and non-coding RNAs expressed in latency IIa, which is associated with HL, employ a variety of mechanisms to target different steps of innate and adaptive immunity, to take advantage of the immunosuppressant effect of immune checkpoints, and to shape the microenvironment to support the survival and proliferation of malignant cells. They suppress the expression or promote the degradation of pattern-recognition receptors, interfere with type I interferon and proinflammatory cytokine mediated signaling, and hinder the effector function of natural killer cells. The processing and presentation of peptides to CD4 and CD8 T cells are also hampered. EBV induces the expression of immune checkpoints, the secretion of immunosuppressive cytokines, and the efflux of regulatory T cells in the tumor microenvironment. The current review provides a comprehensive overview of the molecular mechanisms underlying this complex interplay between EBV and the immune system in HL with focus on clinical data from the pediatric population, which is the key for developing novel, effective therapeutic interventions.

## Simple Summary

Epstein–Barr virus (EBV) is implicated in the pathogenesis of a variety of malignancies, including Hodgkin’s lymphoma (HL). HL is associated with the gene expression program IIa of viral latency, and the expressed proteins and non-coding RNAs mediate both the malignant transformation of B-cells and evasion from immune surveillance. EBV targets pattern-recognition receptors and interferon-mediated signaling to evade innate immunity, hampers the processing and presentation of viral antigen to avoid recognition by CD4+ and CD8+ T cells, induces the expression of immune checkpoint molecules, and shapes the microenvironment to sustain the survival of the malignant cells. The aim of the current review is to shed light on the underlying molecular mechanisms, which is the first step in developing novel effective therapeutic interventions that would harness the immune system against HL.

## 1. Introduction

Approximately 13% of all cancer cases (excluding non-melanoma skin cancers) were attributed to infections in 2018 [1], and according to the International Agency for Research on Cancer, six viruses, namely hepatitis B virus, hepatitis C virus, human papillomavirus, Epstein–Barr virus (EBV), human herpesvirus type 8, also known as Kaposi sarcoma-associated herpesvirus, and human T cell lymphotropic virus type 1, are considered to be carcinogens [2]. Amongst them, EBV undoubtedly holds a special position, as it infects more than 90% of healthy individuals worldwide while being implicated in a wide range of malignancies, including nasopharyngeal carcinoma, Burkitt’s lymphoma and Hodgkin’s lymphoma (HL), diffuse large B cell lymphoma, plasmablastic lymphoma, primary effusion lymphoma, natural killer (NK)/T cell lymphoma, gastric carcinoma, leiomyosarcoma, and post-transplant lymphoproliferative disease [3,4]. EBV is a herpesvirus belonging to the γ-herpesvirus subfamily, also known as human herpesvirus 4, with a linear 172-kbp double-stranded DNA, which encodes approximately 85 proteins and 48 non-coding RNAs [5]. Another significant feature is the virus’s ability to establish a chronic latent infection, during which the viral genome persists in the form of episomes in the nucleus of host cells, and only a subset of the viral genes are expressed [6]. EBV latent infection includes distinct patterns (latency types 0, I, IIa, IIb, and III), each characterized by a specific gene expression signature under strict epigenetic regulation and associated with different EBV-associated malignancies [6]. During the course of EBV infection the pressure of EBV-specific cytotoxic T lymphocytes (CTLs) causes the virus to switch to latency type IIa, a more restricted state in terms of gene expression characterized by the expression of latent membrane proteins 1 and 2A (LMP1 and LMP2A), Epstein–Barr nuclear antigen 1 (EBNA1), and the expression of EBV encoded RNAs (EBER RNAs) and BamHI A rightward transcript (BART) miRNAs [7].

HL is a lymphoid malignancy originating from preapoptotic germinal center B cells [8] with an estimated incidence rate of 25 new cases/million/year in the United States per the Surveillance, Epidemiology, and End Results Program (2000–2022). It exhibits a bimodal distribution curve with the first, larger peak seen in adolescents and young adults (AYAs) [9]. Classical HL (cHL) represents the vast majority of cases. A strong association with EBV infection has been described in approximately 30% of cHL cases, with differences according to age and histological subtype, i.e., the mixed cellularity subtype is the most prevalent in children with a strong association with EBV infection (approximately 80%), while the nodular sclerosis subtype is the most common in older patients and exhibits a weaker association (<30%) [10,11,12]. EBV latency program IIa has been associated with HL, and the expressed genes contribute to HL pathogenesis [7]. LMP1 is a CD40 receptor homolog that stimulates nuclear factor κB (NF-κB) and Janus Kinase/Signal Transducer and Activator of Transcription (JAK/STAT) pathway activation [13,14,15] and shapes the tumor microenvironment (TME) to support the survival of the few Reed–Sternberg (RS) cells [16]. LMP2A substitutes the function of the B cell receptor, thus salvaging the RS cells from apoptosis [17]. EBNA1 is a viral transcription factor that supports the maintenance of EBV episomes and regulates viral and cellular gene expression [18]. HL has a generally favorable prognosis with a five-year relative survival of nearly 90% [9]. However, refractory disease occurs in 5–10% and relapse in 5–30% (depending on stage) of children and adolescents with HL [19].

**Figure 1:**
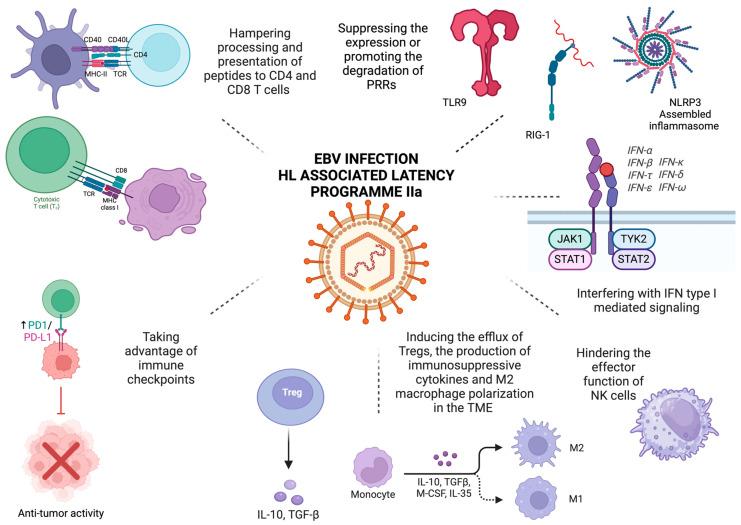
EBV-mediated immune evasion in HL. The proteins and non-coding RNAs expressed in latency IIa, which is associated with HL, target different steps of innate and adaptive immunity, take advantage of the immunosuppressant effect of immune checkpoints, and shape the tumor microenvironment. They suppress the expression of TLR9 by hindering its promoter activity or by suppressing the N^6^-methyladenosine modification and that of NLRP3, thereby blocking the assembly and activation of the NLRP3 inflammasome. They also promote RIG-I degradation through the proteasome pathway, interfere with type I interferon and proinflammatory cytokine-mediated signaling, and hinder the effector function of NK cells. Processing, loading to MHC class I and II, and ultimately recognizing peptides by CD8 and CD4 T cells, respectively, are hampered. Increased expression of immune checkpoint molecules, namely PD-L1 and LAG-3, induces T cell exhaustion and impairs their effector function and thus the clearance of neoplastic cells. Finally, EBV shapes the TM by creating an immunosuppressive niche through attracting Tregs, inducing the expression of regulatory cytokines, such as IL-10 and TGF-β), affecting the polarization of macrophages, and upregulating immune checkpoints. Created in BioRender (accessed on 27 March 2025). Tsotridou, E. (2025).

Immune evasion constitutes one of the hallmarks of cancer [20], and immunotherapies have revolutionized the field of oncology, exhibiting extremely promising results even in heavily pretreated refractory or relapsed cases [21]. Therefore, understanding the complex interplay between known common carcinogens such as EBV, cancer cells, and the immune system constitutes the key to developing novel, effective therapeutic interventions. The current review summarizes the mechanisms underlying EBV-induced immune escape in HL (Figure 1).

## 2. Evading Innate Immunity

Innate immunity constitutes the first line of defense against pathogens activated by pathogen-associated molecular patterns or danger-associated molecular patterns. A plethora of pattern-recognition receptors (PRRs) take part in the initiation of the innate immunity response, including Toll-like receptors (TLRs), RIG-I-like receptors (RLRs), Nod-like receptors, AIM2-like receptors, C-type lectin receptors, and other DNA sensors, such as cyclic GMP-AMP synthase, which activate the NF-κΒ signaling pathway and the production of type I interferons (IFNs) [22,23]. The EBV proteins expressed in latency type II (and subsequently in EBV-associated HL) interfere in different stages of this process in order to escape from immune control.

LMP1 suppresses TLR9 expression by hindering its promoter activity. LMP1 is known to induce NF-κB signaling, which is essential in TLR9 gene downregulation, as the TLR9 promoter contains four NF-κB binding sites [24]. Furthermore, LMP1 promotes RIG-I degradation through the proteasome pathway and specifically by recruiting E3 ligases to mark the protein for degradation by ubiquitination [25]. LMP1 also regulates IFN signaling by interacting with the non-receptor tyrosine-protein kinase Tyk2, a member of the JAK protein family [26]. Type I IFNs exert their function by binding to the heterodimeric Interferon Alpha/Beta Receptor 1/2 (IFNAR1/2). Tyk2 associates with IFNAR1 and, upon activation, induces the phosphorylation of IFNAR1 and recruits STAT1 and 2. STAT1/2 heterodimers, together with IFN-regulatory factor 9, form the IFN-stimulated gene factor 3 complex, which translocates to the nucleus and binds to IFN-stimulated response elements (ISRE) [27]. LMP1 inhibits Tyk2 phosphorylation and, subsequently, STAT2 phosphorylation and ISRE activation [26].

LMP2A/B have been shown to target the same pathway. Although they do not influence the levels of IFN receptors on the cell membrane, LMP2A/B attenuate interferon responses by inducing receptor degradation, either through ubiquitination or through trafficking from endosomes to lysosomes [28].

EBNA1 suppresses N6-methyladenosine modification of TLR9, thus affecting the encoded messenger RNA (mRNA) stability. Mechanistically, EBNA1 promotes the degradation of Methyltransferase 3, N6-Adenosine-Methyltransferase Complex Catalytic Subunit (METTL3), an enzyme catalyzing the formation of N6-methyladenosine, by increasing K48-linked ubiquitination mediated by parkin ubiquitin ligase [29]. EBNA1 targets another vital component of innate immunity, i.e., the function of NK cells [30]. EBNA1 downregulates the expression of UL16 Binding Proteins 1 and 5, both of which serve as Killer Cell Lectin-Like Receptor K1 (NKG2D) ligands. Apart from inhibiting the expression of ligands of NK cell receptors, EBNA1 binds to the c-Myc promoter, which hinders cellular responses to stress and/or DNA damage and apoptosis [30].

The role of EBV proteins expressed in latency IIa in evading innate immunity is summarized in Table 1.

Apart from EBV proteins, several viral miRNAs target PRRs and interfere with IFN- and other proinflammatory cytokine-mediated signaling [31,32,33,34,35,36,37]. miR-BART6-3p targets RIG-I [31,32], while miR-BART15 targets NLR Family Pyrin Domain Containing 3 (NLRP3), the most well-characterized of the Nod-like receptors, thereby inhibiting the production of interleukin 1β (IL-1β) by the inflammasome, as NLRP3 activation results in the assembly and activation of the NLRP3 inflammasome and, in turn, in the release of proinflammatory cytokines [33,38]. Interestingly, this effect was observed both in infected and neighboring non-infected cells, suggesting miRNA trafficking via exosomes [33]. MiR-BART16 targets the histone acetyltransferase cAMP Responsive Element Binding Protein (CREB), which interacts with positive regulatory domains of the IFNβ promoter [34,39]. Skinner et al. demonstrated that the BamHI fragment H rightward open reading frame BHRF1-2-5p blocks IL-1 signaling by targeting receptor 1 of IL-1 (IL1R1) in latently infected B cells [35]. Taking into consideration that this cell surface cytokine receptor initiates a signaling cascade upon activation, which leads to the NF-κB-mediated induction of IL-1α and IL-1β production, BHRF1-2-5p also impacts the expression levels of cytokines [35]. Indeed, upon BHRF1-2-5p inhibition, IL-1β treatment resulted in a two-fold increase in the levels of IL-1α, IL-1β, and IL-6, thus proving the disruption of a positive feedback loop of autocrine/paracrine signaling [35]. Using RNA-induced silencing complex immunoprecipitation followed by polymerase chain reaction target validation, Dölken et al. proved that EBV miR-BART3 targets importin 7, resulting in the reduced production of IL-6 upon lipopolysaccharide challenge, thus also confirming the previous findings of other groups [36,37]. Finally, miR-BART-2p targets MHC Class I Polypeptide-Related Sequence B (MICB), a function preserved amongst herpesviruses [40]. MICB constitutes an NKG2D ligand, which is expressed upon the activation of cellular stress pathways following malignant transformation, thus marking them for recognition by NK cells [41].

## 3. Evading Adaptive Immunity

Although the main EBV proteins that facilitate evasion of adaptive immunity mechanisms are early lytic cycle proteins, the proteins expressed in latency IIa have also been reported to contribute [42].

LMP1 limits its self-presentation to CD8+ T cells [43] and hijacks the cellular transcription program by activating multiple signal transduction pathways, including NF-κB, all three Mitogen-Activated Protein Kinase (MAPK) pathways, namely c-Jun N-terminal kinase, extracellular signal-regulated kinases 1/2 (ERK1/2) and p38, JAK/STAT, and Phosphoinositide 3-kinase/Protein Kinase B (PI3K/Akt) [44]. LMP1 induces the production of IL-10 in B cells [45], which acts not only as an autocrine growth factor for B cells [46] but also as an immunosuppressant [47,48]. Increased IL-10 production results in the downregulation of Antigen Peptide Transporter 1 (TAP1), a part of the heterodimeric complex responsible for peptide transport to the lumen of the endoplasmic reticulum and, subsequently, loading to Major Histocompatibility Complex (MHC) class I molecules [47]. Taking into consideration that molecules lacking loaded peptides also lack stability, it is evident that the reduction in TAP1 expression levels directly influences the steady-state levels of MHC class I molecules [47]. At the same time, increased IL-10 production modifies cell surface glycosylation, thereby increasing the antigenic threshold required for T cell activation [48]. This is achieved through the upregulation of the glycosyltransferase Mgat5, which enhances N-glycan branching on surface glycoproteins, which in turn leads to the formation of a galectin 3-mediated membrane lattice [48]. Mechanistically, LMP1 induces the activation of both PI3K/Akt and p38 pathways through distinct intracellular signaling regions to enhance IL-10 production [45]. P38 mediates the phosphorylation and activation of CREB. The PI3K pathway activation has secondary effects on CREB, as it induces the phosphorylation and inactivation of the Serine/Threonine-Protein Kinase GSK3B, thus hindering its inhibitory effect on CREB-dependent IL-10 production [45]. LMP1 also induces the ectopic expression of CD137 on RS cells and the subsequent secretion of IL-13, which, like IL-10, is a potent growth factor for the neoplastic cells and mediates immune escape by reducing IFNγ production [49,50]. The ectopic expression of CD137 on RS cells, which is a result of the LMP1 mediated activation of the PI3K/Akt/Mechanistic Target of Rapamycin Kinase (mTOR) pathway, eliminates its ligand CD137 from adjacent antigen presenting cells either by internalization and degradation or by trogocytosis, thereby also eliminating the costimulatory signal normally provided by CD137/CD137L signaling for T cell activation [49,50].

LMP2A hinders antigen presentation to CD4+ T cells by reducing the expression of MHC class II molecules [51]. Immunoreceptor tyrosine-based activation motifs within LMP2A interact with the kinases Spleen-Associated Tyrosine Kinase (Syk) and SRC Proto-Oncogene, Non-Receptor Tyrosine Kinase (Src), which mediate the activation of the PI3K/Akt pathway, leading to the reduced promoter activity of E47 and PU.1 [51]. These transcription factors bind to promoter III of the Class II MHC Transactivator, thus regulating its expression levels [51]. There are also reports that LMP2A interferes with the effector function of CD8+ T cells through multiple mechanisms: it downregulates (to a lesser extent) the expression of MHC class I molecules, reduces the recognition of EBV+ neoplastic B cells by reducing the expression of NKG2D ligands, and finally, again through the initial activation of Syk, leads to the activation of PI3K and Bruton kinase-mediated signaling to induce the phosphorylation of STAT3 and ultimately the production of IL-10 [52,53].

EBNA1 acts as an inhibitor of both ribosomal and proteosomal activity, thereby preventing the presentation of its epitopes by MHC class I molecules and their recognition by CD8+ T cells. The glycine-alanine repeat domain (GAr) within EBNA1 acts in cis and inhibits the translation of its own mRNA [54,55,56]. Interestingly, this effect is location-dependent since it is required for the GAr domain to be located within the coding sequence of the gene to have an effect on the process of translation [55]. EBNA1 has also been reported to inhibit its own degradation by interfering with the initial steps of substrate unfolding [57]. The extent to which each process contributes to immune evasion remains unclear, although there are reports that the inhibition of translation is sufficient to prevent the presentation of viral peptides to cytotoxic cells [56]. However, it should be noted that a low turnover rate is also required for a low rate of protein synthesis.

The role of EBV proteins expressed in latency IIa in evading adaptive immunity is summarized in Table 2.

The role of EBV miRNAs in evading adaptive immunity was initially demonstrated by experiments with miRNA-deficient EBV strains. Upon the in vivo infection of mice with reconstituted human immune system components with EBV strains expressing or lacking miRNAs, the absence of miRNAs resulted in reduced viral loads, lymphomagenesis, and T cell proliferation [58]. However, antibody-mediated CD8+ T cell depletion caused an increase in viral loads, which was more evident in miRNA-deficient (200-fold increase) compared to wild-type EBV-infected cells (40-fold increase) [58]. Moreover, more than 50% of animals developed tumors, while no tumors were reported prior to CD8+ T cell depletion in the miRNA-deficient group [58]. These results suggest that EBV-encoded miRNAs mediate immune evasion by hindering the clearance of EBV-infected B cells by CD8+ T cells, thus also contributing to lymphomagenesis [58]. EBV miRNAs target LMP1 and interfere with the release of proinflammatory cytokines, antigen processing, and presentation either by MHC class I or class II molecules [59,60]. MiR-BART3 and miR-BART16 directly target LMP1, thus limiting its expression and, consequently, the EBV-induced antigenic stimuli [59]. The production of proinflammatory cytokines is also hindered [59]. Particularly, IL-12, which is targeted by miR-BART1, miR-BART2, miR-BART10, miR-BART22 and BHRF1-2, plays a critical role in the differentiation of CD4+ to Th1 T cells [59,60]. MiR-BART17 and BHRF1-3 target Antigen Peptide Transporter 2 (TAP2), a part of the heterodimeric complex responsible for peptide transport and loading to MHC class I molecules [60]. Several EBV miRNAs also target cathepsin B (CTSB), Lysosomal Thiol Reductase (IFI30), and Asparaginyl Endopeptidase (AEP), lysosomal enzymes involved in antigen processing and MHC class II-mediated presentation [59,60].

The miRNAs, along with their targets implicated in evasion of both innate and adaptive immunity, are presented in Table 3.

## 4. Taking Advantage of Immune Checkpoints

Programmed death receptor 1 (PD-1) is a transmembrane glycoprotein that is expressed on the surface of activated CD4+ and CD8+ T lymphocytes, B lymphocytes, dendritic cells (DCs), and NK cells [61,62]. PD-1 activation via binding of its PD-L1 and PD-L2 ligands inhibits T cell growth, proliferation, and effector functions via the dephosphorylation of essential components of the T cell receptor (TCR) signaling cascade, the inhibition of the activation of the PI3K/Akt/mTOR and Ras/MEK/Erk pathways, and the hampering of T cell motility and interaction with antigen presenting cells [61,62]. PD-1 signaling eventually inhibits the entry of T cells into the S phase of the cell cycle, causes a metabolic shift towards increased fatty acid oxidation, and increases the differentiation of T cells into induced regulatory T cells (Tregs) [61,62]. According to a recent meta-analysis, the expression levels of PD-L1 were higher not only on tumor cells but also on immune cells in the TME in EBV-positive compared to EBV-negative cases of cHL, with risk ratios of 1.66 and 1.43, respectively [63]. Alterations of 9p24.1/CD274(PD-L1)/PDCD1LG2(PD-L2), which constitute a defining feature of cHL and increase PD-L1 or PD-L2 expression, have been shown to be similarly distributed in patients with EBV-negative and EBV-positive cHL, but EBV-positive cHLs displayed higher PD-L1 H-scores (percentage of RS cells with positive staining multiplied by the average intensity of positive staining), suggesting further induction of PD-L1 expression by EBV infection [64]. Mechanistically, EBV LMP1 induces PD-L1 expression in two ways: JAK/STAT signaling mediated enhanced promoter activity and activator protein 1-mediated enhancer activity [65,66]. Of note, the LMP1-associated induction of PD-L1 is fine-tuned by viral miRNA BHRF1-2-5p, which binds to the 3’untranslated region, thus inhibiting gene expression [67]. Carey et al. demonstrated that PD-L1 is mostly expressed by tumor-associated macrophages (TAMs) and that these PD-L1-positive TAMs tend to colocalize with PD-L1-positive RS cells. The vicinity of this cellular niche is enriched with PD-1-positive T cells, thus facilitating T cell exhaustion and immunosuppression [68].

Cytotoxic T lymphocyte-associated protein 4 (CTLA-4) is a CD28 homolog. Binding to its ligands, CD80 and CD86, inhibits their interaction with CD28 and thus the necessary co-stimulatory signal in the immune synapse for T cell activation [69]. Evidence regarding the role of CTLA-4 in EBV-related malignancies and particularly Hodgkin’s lymphoma remains scarce. However, data from a cord blood-humanized mouse model suggest that both PD-1 and CTLA-4 are expressed on T cells and that PD-1/CTLA-4 blockade increases T cell infiltration of tumors, promotes T cell activation, and eventually leads to a drastic reduction of the size of EBV-induced lymphomas [70].

Other immune checkpoints, including the protein encoded by lymphocyte activation gene-3 (LAG-3), T cell immunoglobulin-3 (TIM-3), T cell immunoglobulin and ITIM domain (TIGIT), V-Domain Ig suppressor of T cell activation (VISTA), the B7 homolog 3 protein (B7-H3), the B and T cell lymphocyte attenuator (BTLA), and the sialic acid-binding immunoglobulin-like lectin 15 (Siglec-15), are constantly gaining attention. However, questions about their ligands and mechanisms of action still remain, and there are limited preclinical and clinical data [62].

LAG-3 is expressed on CD4+ and CD8+ T cells, regulatory T cells (T-regs), a subpopulation of NKs, B cells, and plasmacytoid DCs and binds to MHC class II with a 100-fold higher affinity than CD4, thus hampering TCR-mediated signaling [71]. Gandhi et al. demonstrated that LAG-3 is expressed on tumor-infiltrating lymphocytes adjacent to the malignant RS cells and that higher expression levels correlate with EBV positivity. Furthermore, they reported that the immunological responses against LMP1 epitopes are impaired in newly diagnosed or relapsed HL patients and that the observed T cell functional impairment is proportional to the degree of LAG-3 and forkhead box protein P3 (FOXP3), a marker of Tregs. These results suggest a significant role of LAG-3-expressing Tregs in the microenvironment of EBV-positive RS cells in dampening T cell responses and leading to immune evasion [72].

Specifically in the pediatric population, Dilly-Feldis et al. assessed ligand PD-L1 in 42 children with cHL and reported higher expression levels in EBV-positive cases [73]. Based on these findings, Uccini et al. used immunostaining to measure the expression of PD-1 and PD-L1 in 53 cases of cHL in children under 14 years of age and, apart from confirming that PD-L1 levels are higher in EBV cases, proved that increased PD-L1 expression is independent of 9p24.1 amplification, as all of the EBV positive cases were negative for 9p24.1 amplification by fluorescent in situ hybridization [74]. Recently, Oscar et al. demonstrated that LAG-3 positive cells have a positive correlation with PD-1 positive cells in cases of cHL, which remains significant when analyzing exclusively EBV positive cases, but is lost in EBV negative ones [75]. EBV-positive cases with LAG-3 and PD-1 co-expression had strikingly lower 5-year survival rates compared to either LAG-3 and PD-1 negative cases or cases with expression of one of the exhaustion markers (54% versus 100%) [75].

## 5. Shaping the Microenvironment

Despite the fact that RS cells constitute the defining feature of HL, they comprise only 1% of the tumor, while the rest represents a plethora of immune cells including T and B lymphocytes, neutrophils, eosinophils, macrophages, plasma cells, NK cells, dendritic cells and mast cells, as well as stromal cells, fibroblasts, and endothelial cells. This diverse cellular infiltrate is in a continuous crosstalk with the few neoplastic cells and plays a pivotal role in sustaining tumor growth and facilitating immune escape [76,77,78]. The significance of the composition of the TME is evident by its great prognostic and predictive value in terms of response to treatment and survival [79,80]. EBV seems to play a unique role in shaping the TME and creating an immunosuppressive niche by attracting Tregs, inducing the expression of regulatory cytokines, affecting the polarization of macrophages, and upregulating immune checkpoints [65,66,81,82,83,84,85,86,87,88,89]. Morales et al. compared gene expression in the lymph nodes and the peripheral blood between EBV-positive and -negative HL patients aged between 8 and 71 years. In this mixed-age patient population, EBV-positive cases were characterized by an upregulation of CD4, FOXP3, which is a marker of Tregs, adhesive molecules, such as integrin B2 and p-selectine, and immune checkpoints, such as CTLA-4 and LAG-3 [82]. Furthermore, the increased expression of immunosuppressive cytokines (IL-10) and transforming growth factor beta (TGF-β) was detected both in the lymph nodes and the sera of EBV-positive patients [82]. The efflux of Tregs was attributed to an increased production of cc motif chemokine (CC) ligands 4, 5, 17, 19, and 20 and their receptors CCR5 and CCR7 [82]. A distinct subpopulation of Tregs, namely T regulatory 1 cells, secreted IL-10 both in the lymph nodes and the periphery [82], which has also been confirmed by other groups [83,84]. EBV-encoded proteins LMP1 and EBNA1, by upregulating CCL20, have been reported to attract Tregs in the TME [83,85,90]. Furthermore, stromal cells in the TME express indoleamine 2,3-dioxygenase (IDO), an enzyme that suppresses T cell effector functions while at the same time enhancing the immunosuppressive effect of Tregs [91,92]. Higher IDO expression has been reported in EBV-positive cases and has been associated with inferior survival [91]. The composition of the TME also differs between adults and children and even varies among different age groups in the pediatric population [93]. Interestingly, Barros et al. demonstrated that in the pediatric population, EBV positivity was not associated with increased infiltration of FOXP3 cells, but rather CD8, Cytotoxic Granule-Associated RNA Binding Protein TIA1, Granzyme B, and T-Box Transcription Factor 21-positive cells, suggesting a cytotoxic Th1 profile [93]. This is further reinforced by the distinct macrophage composition and their M1 polarization in young children with EBV-associated HL, which greatly influences survival rates [88,89,94]. However, Jimenez et al. reported the recruitment of PD-L1-positive cells in the microenvironment of pediatric HL cases [81]. Despite the presence of cytotoxic T lymphocytes [86,87,93] both in pediatric and adult patients, the concomitant upregulation of immune checkpoint molecules [65,66,81] probably induces exhaustion and hinders their effector functions.

## 6. Conclusions

EBV latency program II is associated with Hodgkin’s lymphoma, and the virus plays a critical role not only in malignant transformation but also in the maintenance of the neoplastic cells by facilitating escape from anti-tumor immune control. The proteins and non-coding RNAs expressed target all the frontiers of the immune response to support the survival of the malignant cells [95]. The current review provides a comprehensive overview of the molecular mechanisms underlying this complex interplay. Understanding these mechanisms would be the first step in the process to develop novel therapeutic interventions, which would harness the immune system to clear the neoplastic cells. Immune checkpoint inhibitors have already gained approval and proven their efficacy both in the adult [96,97] and in the pediatric population [98,99]. Strategies, such as the use of EBV-specific CTLs in the autologous [100,101,102] or the allogeneic setting [103], EBNA-1 targeted inhibitors [104], and Chimeric Antigen Receptor T cells are currently being investigated [105,106,107]. Creating an EBV-targeted vaccine has been a long-lasting effort, with research focusing on the gp340 glycoprotein [108]. Combining agents that target different evasion mechanisms aimed at different steps of the immune response could provide a promising alternative. However, this course of action also requires attention to the possibility of off-target effects and increased associated toxicities. Children and adolescents undoubtedly constitute a diverse and unique patient population, especially in the context of immunotherapy. There is a pressing need for new clinical trials in HL with different age groups of the so-called pediatric population to explore the potential of novel immunotherapeutics.

## Figures and Tables

**Table 1:** The role of EBV proteins expressed in latency IIa in evading innate immunity.

EBV Protein	Target/Interaction Partner	Molecular Mechanism	References
LMP1	TLR9	NF-κB-mediated suppression of promoter activity	[24]
	RIG-I	Ubiquitination and proteasome-mediated degradation	[25]
	Tyk2	Inhibition of Tyk2 and, subsequently, STAT2 phosphorylation and ISRE activation (necessary for Type I IFN-mediated signaling)	[26]
LMP2A/B	IFN receptors	Degradation through ubiquitination or through trafficking from endosomes to lysosomes	[28]
EBNA1	TLR9	Degradation of METTL3, suppression of N^6^-methyladenosine modification, and reduction of TLR9 mRNA stability	[29]
	UL16 Binding Proteins 1 and 5	Inhibition of NK cell receptor ligands	[30]
	c-Myc promoter	Inhibition of cellular responses to stress and/or DNA damage and apoptosis	[30]

**Table 2:** The role of EBV proteins expressed in latency IIa in evading adaptive immunity.

EBV Protein	Target/Interaction Partner	Molecular Mechanism	References
LMP1	TAP1	IL-10 mediated downregulation resulting in impaired peptide loading to MHC class I molecules and reduced MHC class I levels	[47]
	MGAT5	IL-10 mediated upregulation resulting in modification of cell surface glycosylation and increase in the antigenic threshold required for T cell activation	[48]
	CD137 (ectopic expression on RS cells)	IL-13 secretion and reduction of IFNγ production, elimination of costimulatory signal for T cell activation	[49,50]
LMP2A	Syk, Src	Activation of PI3K/Akt pathway, reduced promoter activity of E47 and PU.1, and subsequently reduced MHC class II expression	[51]
	Syk	Activation of PI3K and Bruton kinase and increased IL-10 production	[52]
	NKG2D ligands	Downregulation resulting in reduced recognition of EBV+ neoplastic B cells	[53]
EBNA1	NA	Inhibition of ribosomal and proteosomal activity and thereby MHC class I presentation, inhibition of “self” translation and degradation	[54,55,56]

**Table 3:** MiRNAs and their targets implicated in evasion of innate and adaptive immunity.

EBV Encoded miRNA	Target	Function	References
miR-BART1	IL-12	Block of differentiation of CD4+ to Th1 T cells	[59,60]
	IFI30	Inhibition of antigen processing	[59,60]
miR-BART2 (5p or 3p)	MICB	Inhibition of recognition by NK cells	[40]
	IL-12	Block of differentiation of CD4+ to Th1 T cells	[59,60]
	CTSB	Inhibition of antigen processing	[59,60]
	AEP	Inhibition of antigen processing	[59,60]
miR-BART3	Importin 7	Reduced production of IL-6	[36,37]
	LMP1	Limiting antigenic stimuli	[59]
miR-BART6-3p	RIG-1	Inhibition of viral recognition and subsequent induction of IFN type I response	[31,32]
miR-BART10	IL-12	Block of differentiation of CD4+ to Th1 T cells	[59,60]
miR-BART15	NLRP3	Inhibition of assembly and activation of the NLRP3 inflammasome and subsequent IL-1β production	[33,38]
MiR-BART16	CREB binding protein	Inhibition of the positive regulatory effect on the IFNβ promotor	[34,39]
	LMP1	Limiting antigenic stimuli	[59]
MiR-BART17	TAP2	Peptide transport and loading to MHC class I molecules	[60]
miR-BART22	IL-12	Block of differentiation of CD4+ to Th1 T cells	[59,60]
BHRF1-2	IL1R1	Blocking IL-1 signaling	[35]
	IL-12	Block of differentiation of CD4+ to Th1 T cells	[59,60]
	CTSB	Inhibition of antigen processing	[59,60]
BHRF1-3	TAP2	Peptide transport and loading to MHC class I molecules	[60]

## Data Availability

Not applicable.

## Abbreviations

The following abbreviations are used in this manuscript:

EBV	Epstein–Barr virus
HL	Hodgkin’s lymphoma
NK	Natural killer
CTLs	Cytotoxic T lymphocytes
LMP1	Latent membrane protein 1
LMP2A	Latent membrane protein 2A
EBNA1	Epstein–Barr nuclear antigen 1
EBERs	EBV-encoded RNAs
BART	BamHI A rightward transcript
AYAs	Adolescents and young adults
cHL	classical HL
NF-κB	Nuclear factor κB
JAK	Janus kinase
STAT	Signal Transducer and Activator of Transcription
TME	Tumor microenvironment
RS	Reed–Sternberg
PRRs	Pattern-recognition receptors
TLRs	Toll-like receptors
RLRs	RIG-I-like receptors
IFNs	Interferons
IFNAR1/2	Interferon Alpha/Beta Receptor 1/2
ISRE	IFN-stimulated response elements
mRNA	messenger RNA
METTL3	Methyltransferase 3, N6-Adenosine-Methyltransferase Complex Catalytic Subunit
NKG2D	Killer Cell Lectin-Like Receptor K1
NLRP3	NLR Family Pyrin Domain Containing 3
IL-1β	Interleukin 1β
CREB	cAMP Responsive Element Binding Protein
BHRF	BamHI fragment H rightward open reading frame
IL1R1	Receptor 1 of IL-1
MICB	MHC Class I Polypeptide-Related Sequence B
MAPK	Mitogen-Activated Protein Kinase
ERK	Extracellular signal-regulated kinases
PI3K	Phosphoinositide 3-kinase
Akt	Protein Kinase B
TAP1/2	Antigen Peptide Transporter 1/2
MHC	Major Histocompatibility Complex
mTOR	Mechanistic Target of Rapamycin Kinase
Syk	Spleen-Associated Tyrosine Kinase
Src	SRC Proto-Oncogene, Non-Receptor Tyrosine Kinase
GAr	Glycine-alanine repeat
CTSB	Cathepsin B
IFI30	Lysosomal Thiol Reductase
AEP	Asparaginyl Endopeptidase
PD-1	Programmed death receptor 1
PD-L1/2	Programmed death receptor ligand 1/2
DCs	Dendritic cells
TCR	T cell receptor
Tregs	Regulatory T cells
TAMs	Tumor-associated macrophages
CTLA-4	Cytotoxic T lymphocyte-associated protein 4
LAG-3	Lymphocyte activation gene-3
TIM-3	T cell immunoglobulin the (BTLA) and the (Siglec-15)
TIGIT	T cell immunoglobulin and ITIM domain
VISTA	V-Domain Ig suppressor of T cell activation
B7-H3	B7 homolog 3 protein
BTLA	B and T cell lymphocyte attenuator
Siglec-15	sialic acid-binding immunoglobulin-like lectin 15
FOXP3	Forkhead box protein P3
TGF-β	Transforming growth factor beta
CC	cc motif chemokine
CCR	cc motif chemokine receptor
IDO	Indoleamine 2,3-dioxygenase
